# Probing Defects in Covalent Organic Frameworks

**DOI:** 10.1021/acsami.4c12069

**Published:** 2024-09-16

**Authors:** Saba Daliran, Ali Reza Oveisi, Amarajothi Dhakshinamoorthy, Hermenegildo Garcia

**Affiliations:** † Department of Organic Chemistry, Faculty of Chemistry, Lorestan University, Khorramabad 68151-44316, Iran; ‡ Department of Chemistry, University of Zabol, P.O. Box: 98615-538, Zabol, 98613-35856, Iran; § Departamento de Química, Universitat Politècnica de València, C/Camino de Vera, s/n, 46022, Valencia, Spain; ∥ School of Chemistry, Madurai Kamaraj University, Madurai 625 021, Tamil Nadu, India; ⊥ Instituto de Universitario de Tecnología Química (CSIC-UPV), Universitat Politècnica de València, Av. de los Naranjos, 46022, Valencia, Spain

**Keywords:** Covalent Organic Frameworks, Defect engineering, Defect characterization, Disorders, Imperfections, Missing-linker structures, Modulated synthesis, Truncation strategy

## Abstract

Defects in covalent organic frameworks (COFs) play a pivotal role in determining their properties and performance, significantly influencing interactions with adsorbates, guest molecules, and substrates as well as affecting charge carrier dynamics and light absorption characteristics. The present review focuses on the diverse array of techniques employed for characterizing and quantifying defects in COFs, addressing a critical need in the field of materials science. As will be discussed in this review, there are basically two types of defects referring either to missing organic moieties leaving free binding groups in the material or structural imperfections resulting in lower crystallinity, grain boundary defects, and incomplete stacking. The review summarizes an in-depth analysis of state-of-the-art characterization techniques, elucidating their specific strengths and limitations for each defect type. Key techniques examined in this review include powder X-ray diffraction (PXRD), infrared spectroscopy (IR), thermogravimetric analysis (TGA), nuclear magnetic resonance (NMR), X-ray photoelectron spectroscopy (XPS), scanning electron microscope (SEM), scanning transmission electron microscopy (STEM), scanning tunneling microscope (STM), high resolution transmission electron microcoe (HRTEM), gas adsorption, acid–base titration, advanced electron microscopy methods, and computational calculations. We critically assess the capability of each technique to provide qualitative and quantitative information about COF defects, offering insights into their complementary nature and potential for synergistic use. The last section summarizes the main concepts of the review and provides perspectives for future development to overcome the existing challenges.

## Graphical abstract

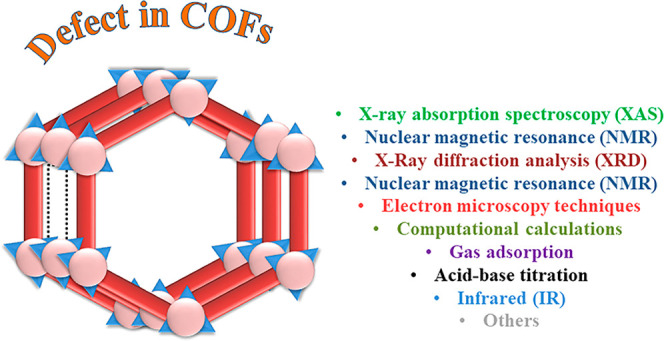

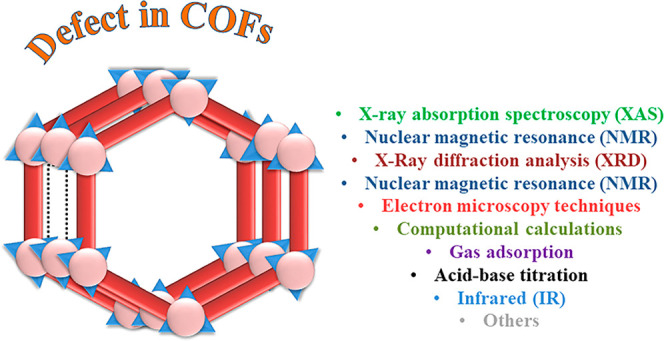

## 1 Introduction

Covalent organic frameworks (COFs) are crystalline porous materials constituted exclusively by organic moieties connected through covalent bonds.^1–5^ One of the important features of COFs is that they are constituted by light elements like C, H, O, N and B and the units are interconnected to form either two-dimensional (2D) or three-dimensional (3D) structures in an extended manner. The construction of a 2D or 3D COF network depends on the geometries of the molecular precursors and their ability to form planar stacked or nonplanar interconnected structures.^6–9^ COFs have attracted considerable attention over the past two decades owing to their intriguing properties including high porosity, large surface area, high chemical/thermal stability, chemical and structural versatility, low density, and potential for facile surface functionalization and postsynthetic modification (PSM).^10–17^ In general, COFs are mostly synthesized through the reversible condensation of building blocks resulting long-range-ordered highly crystalline structures with pores allowing mass transfer from the exterior to the interior of the particles.^18,19^ The better chemical stability of COFs in comparison with related materials such as metal–organic frameworks (MOFs) is attributed due to their metal-free nature and purely covalently bonded nature.^20,21^

In addition, stacking interactions and hydrogen bonds strengthen the porous skeletons of the COFs from hydrolysis and solvation. Owing to these advantageous properties, COFs have been extensively employed as insoluble solid materials in different fields like gas adsorption, separation, catalysis, sensing, biomedicine, energy storage, ion conduction and so on.^22–33^ For most of these applications, defects play crucial roles.

In summary, defects can consist in the absence of one of the organic moieties, leaving an incomplete binding position in the neighbor complementary monomers, the presence of some comonomer with lesser connectivity, but they can also be imperfections in the crystal structure or in the stacking and folding of the polymeric framework.^34–36^ When a linker/knot vacancy is present, the physical/chemical properties of the unsaturated groups near the position can determine the performance of the defective COF in most of the applications. For instance, these free functional groups can interact with gases, increasing the adsorption capacity of the material and/or altering the gas permeation selectivity.^37,38^ Crystallinity imperfections affect the surface area and pore size distribution of COFs, which are crucial parameters influencing their performance in various applications.^39–43^ Indeed, COFs can exhibit several types of defects (Figure 1), which include the following:

1. Missing linkers/knots:^35,36,43,44^ Absence of organic building blocks in the framework, creating vacancies or dangling bonds. These can be further considered as vacancy (point) deects in crystallography. It is different from bonding surface defects (as intrinsic bonding defects) which are the unbounded functional groups at the end of COF, including dangling bonds.^45–47^

2. Imperfections in the crystal structure can be further categorized into:

2a. Dislocations and distortions: The atoms or molecules are dislocated from their ideal lattice structure along a line, creating a line of misalignment.^48^ They can also related to deviations from the ideal crystal structure due to strain or imperfections.^41,49,50^

2b. Defects include grain boundaries (interfaces between different crystalline domains within the COF)^51–54^ and stacking disorders (irregularities in the way 2D COF layers stack on top of each other).^39,55–59^

2c. Low crystalline regions: Areas within the COF lacks long-range order.^60–62^

Dislocations and grain boundaries can be considered as topological defects and irregularities in the connectivity of the monomers and oligomers.^63,64^

**Figure 1:**
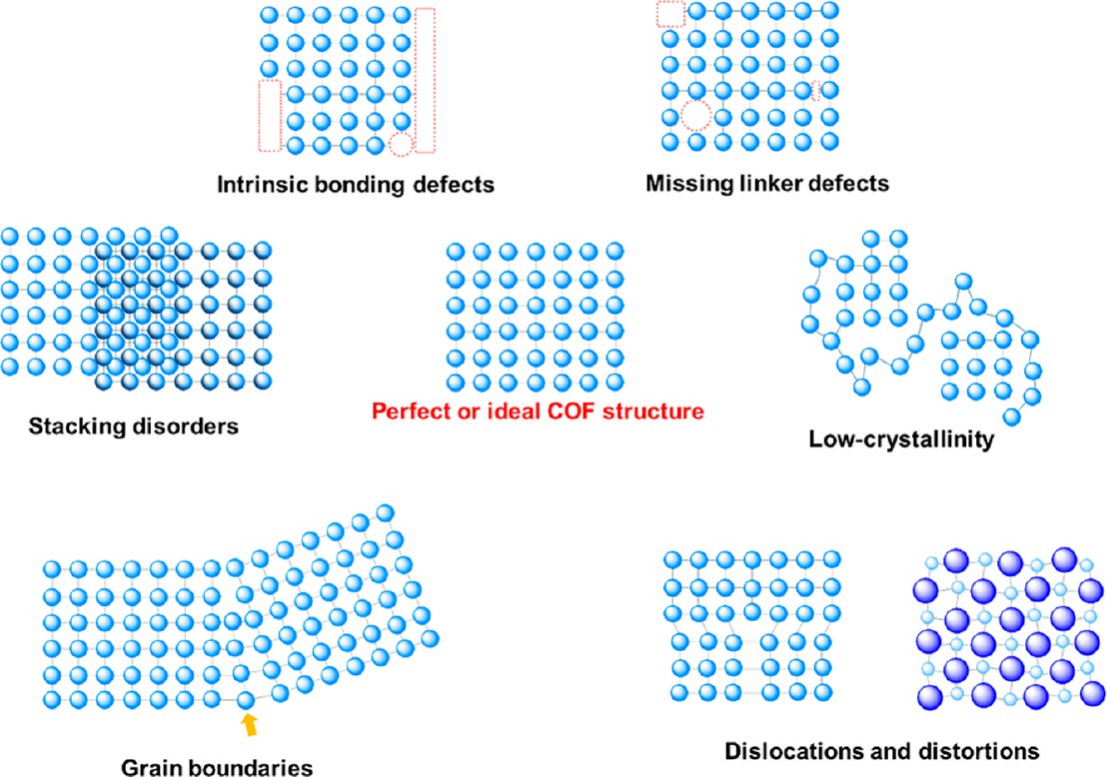
Types of defects in the COFs. The components are represented as follows: spheres symbolize the organic nodes, while lines represent the organic linkers that connect these nodes. Together, these two components form the building blocks of the COF structure. Empty boxes/circles or missing connections highlighted in red indicate intrinsic bonding defects or missing-linker defects in the COF framework.

The origin of defects can be traced as derived from various factors in the COF synthesis process, including crystallization rate, purity, shape, molar ratios, and relative precursor reactivity, synthetic conditions (temperature, pressure, solvent, etc.),^44^ the use of end-capping monomer/modulator,^65–68^ monomer-symmetry regulation strategy,^69,70^ template strategy,^71^ and postsynthetic treatments.^65^ Understanding the nature and origin of defects is crucial to selecting appropriate characterization techniques and interpreting the results accurately. The present review focuses on experimental techniques able to provide information on the existence of these defects and on their quantification. This is a challenging issue because defects are generally present in the material in a minor percentage and can have different origins and nature. Throughout the review, the various analytical and spectroscopic techniques suitable for the defect characterization will be commented on, making emphasis on their suitability for specific defect types and their limitation, particularly regarding quantification of their concentration. A general idea that will be conveyed is that the characterization of defects, being a complex problem, requires the combination of several of these techniques and the comparison among different samples. In a certain way, it will become evident that these techniques provide an indication of the presence and relative density of defects by comparing several samples, in most cases considering one of them as ideally defect-free. It will remark that some of these advanced techniques provide better evidence and detailed information about the nature and location of the defective sites.^36,72^ In the case of transmission electron microscopy (TEM) with *quasi* atomic resolution, defects in COFs can be visualized, but analysis requires performing a careful statistical study of their distribution and number throughout various regions of the sample. Solution NMR spectroscopy of digested COFs provides crucial insights into defect sites and surface functionalities. This approach involves carefully dissolving the COF structure in appropriate solvents, allowing for detailed analysis of its components.

As will be emphasized in the conclusions, understanding the nature and density of defects in the COFs is crucial. This information allows us to better comprehend the behavior of the COF in various applications. Additionally, this helps rationalize, at the molecular and structural level, how these polymeric materials function as catalysts. These insights are the primary motivation for thoroughly characterizing defects in the COFs.

## 2 Identifying and Quantifying Defects

While XRD can report at a glance on the crystalline structure of a solid, detection and characterization of atomic-level defects in COFs still remains a challenge, because direct probing of local structures in these materials frequently requires access to advanced characterization techniques. The nature and dimensions of the local defects are generally not reported by routine characterization techniques, which have limited resolution and usually give the average properties of the materials. In these routine techniques, the presence of defects in COFs is deduced indirectly without the real capability to monitor them directly. Most commonly, the presence, nature, and density of defects in COFs are presumed from the combination of data from several conventional techniques, including IR and NMR spectroscopy, TGA, gas adsorption–desorption experiments, acid–base titration, single-crystal X-ray diffraction (SC-XRD, SXRD, or SCD), PXRD, X-ray absorption spectroscopy (XAS), dynamic laser scattering (DLS), XPS, SEM, TEM, STEM, STM, and even computational calculations. The following table (Table 1) and sections will summarize how these techniques can report on the defects present in the COF materials.

**Table 1:** Summary of the Different Techniques Used to Identify Defects in COFs

Technique	Quantitative/Qualitative	Principle/Operational mode	Defects Detected	Advantages	Limitations	ref
IR	Qualitative/Semiquantitative	Vibrational spectroscopy	Missing linkers, surface defects, functional groups	Rapid and simple sample preparation	Limited to IR-active functional groups as defects	^35,36,45,70,73^
NMR	Quantitative	Nuclear spin interactions	Local chemical environment, missing linkers	Detailed structural information	Require careful sample dissolution	^36,44,74,75^
TGA	Qualitative	Thermal decomposition	Mostly not limited to missing linkers (and guest molecules)	Quantifies thermal stability, simple operation	Limited to thermally induced changes	^36,76^
Gas sorption	Qualitative	Gas–solid interactions	Crystallinity, stacking disorders, missing linkers	Provides surface area and pore size distribution, nondestructive analysis	Indirect defect detection	^35,36,43,74,77^
Acid–base titration	Quantitative	Acid–base reaction	Missing linkers, surface defects	Does not require special or expensive chemicals	Limited by the accessibility of the defect sites to the titrant	^74,78^
XRD	Qualitative	Diffraction of X-rays	Crystallinity, stacking disorders, and layer distances	Nondestructive, bulk analysis	Limited information on local defects	^35,36,43,56,57,79,80^
XPS	Quantitative/Qualitative	Photoelectron spectroscopy	Surface and missing-linker defects, chemical environment, elemental composition	Surface-sensitive, chemical state information	Limited depth analysis without profiling	^35,36,43,81^
TEM/HRTEM (and STEM)	Qualitative	Electron interactions (Focused electron beam scanning)	Lattice distortions, dislocations and stacking faults, grain size and boundaries, quantifying the offset between layers	Direct visualization of defects (High resolution, element mapping, characterizing beam-sensitive materials)	Sample preparation challenges, potential beam damage, small sample area (andits limited availability)	^51,52,54,82–86^
STM	Qualitative	Quantum tunneling	Surface defects, atomic-scale features, grain boundary defects	Atomic resolution of surface structures	Limited to conductive samples, surface-only analysis	^64,87^
Computational calculations	Quantitative/Qualitative	Theoretical modeling	Various defect types, energetics	Predicts defect formation, properties	Dependent on model accuracy, computationally intensive	^39,48,53,63,88,89^

### 2.1 IR Spectroscopy

IR spectroscopic techniques, particularly Fourier transform IR (FT-IR), can be used to monitor the presence of free bonding functional groups or dangling linkers in defective COFs, as well as their PSM.^70,90,91^ For example, Guan and co-workers showed the presence of residual −CHO functional groups that should have reacted completely in an imine-linked COF, TPB-DMTP COF (**1**) (TPB: trisaminophenylbenzene, DMTP: 2,5-dimetoxyterephthaldehyde) (Figure 2). −CHO groups were characterized by recording their characteristic FT-IR stretching band at 1682 cm^–1^.^45^ Besides, it was also reported that the IR spectroscopy also supports postsynthetic derivatization of COF (**1**) with the mono aminoporphyrin molecule ((5-(4-aminophenyl)-10,15,20-triphenylporphyrin, Por)) decorating the external surface, via a Schiff-base condensation and, afterward, the loading of vanadyl-naphthalocyanine (VONc) guest via soaking in a polar solvent, thereby confirming the successful formation of COF-Por (**2**) and VONc@COF-Por (**3**), respectively. In this series of reactions, IR spectra show the appearance of the stretching modes of in-plane Ar–H (∼790 cm^–1^) and pyrrole N–H (∼3320 cm^–1^) along with the disappearance of the stretching vibrations of the -NH_2_ groups (∼3640 and 3540 cm^–1^) for the first step going from **1** to **2** and the presence of V=O (∼1010 cm^–1^) and aliphatic C–H groups (∼2960 and 2870 cm^–1^) for the last step going from **2** to **3**. It should be noted that a characteristic peak of the imine linkages in the three imine-based COFs around 1620 cm^–1^ remained intact. They considered the nanoscale COFs for biomedical applications.

**Figure 2:**
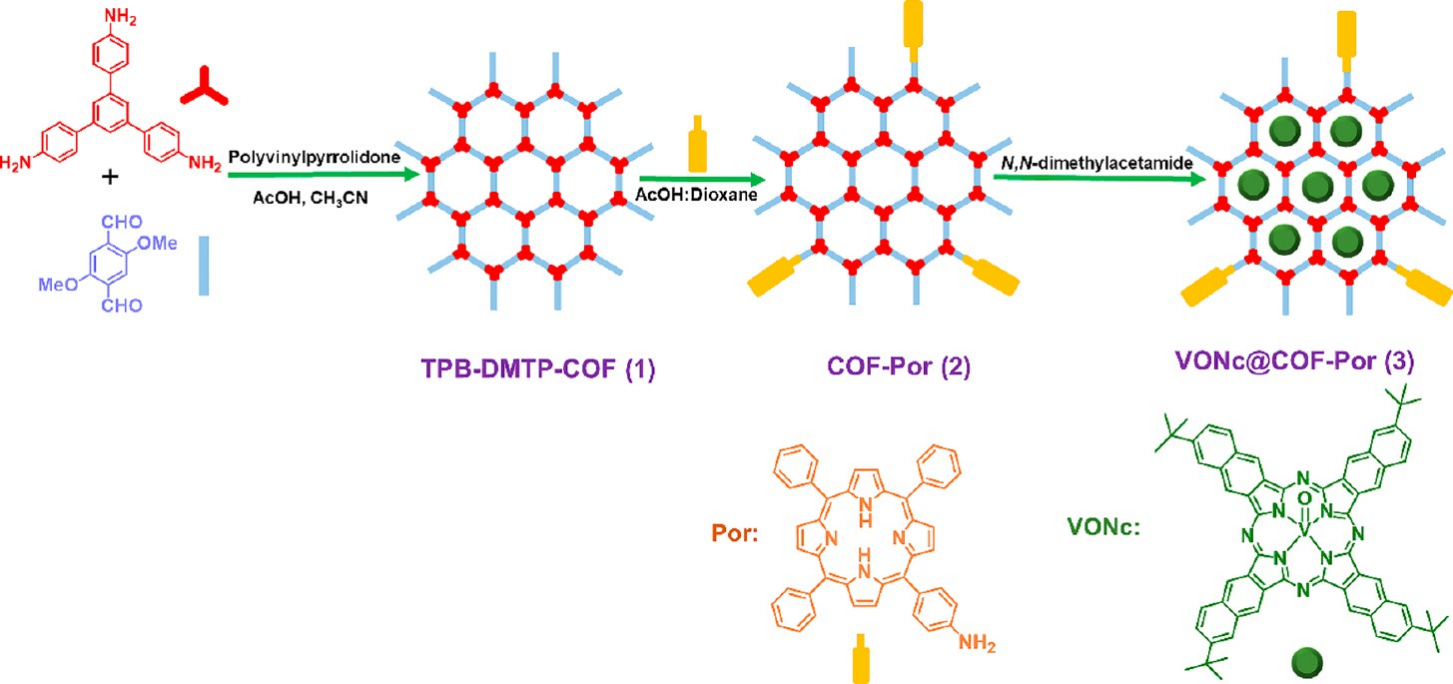
Schematic Synthesis of VONc@COF-Por. Reproduced from ref. [^45^]. Copyright 2019 American Chemical Society.

Defect introduction in COFs has been widely achieved through monofunctional truncated monomer or modulator-assisted synthesis.^65^ Jiang and co-workers have shown the formation of defective COF nanosheets (Figure 3a), termed NCOFN-X (N refers to a defective COF with excess −NH_2_ groups, COFN stands for COF nanosheets, X (10, 30, or 50) represents the percentage of monoaldehyde end-capping units relative to total aldehyde components and calculated as [DHB]/[DHB + DHA (or DHTA)] × 100, where DHB (2,5-dihydroxybenzaldehyde) is the monoaldehyde end-capping unit and DHA or DHTA (2,5-dihydroxyterephthalaldehyde) is the dialdehyde building unit), which acted as efficient CO_2_ separation materials.^35^ The DHB value controls the degree of defects introduced into the COF structure, with higher X values corresponding to more defects and unreacted amine groups of the amine monomer in the framework. The imine-linked NCOFN is synthesized via a condensation reaction involving a mixture of dialdehyde (DHA or DHTA) and monoaldehyde (DHB) with an amine monomer (triaminoguanidinium, TG_Cl_). The introduction of monoaldehyde as an end-capping monomer during COF assembly leads to the *in situ* formation of missing-linker defects and introduction of unreacted amino groups from TG_Cl_ as dangling sites.

**Figure 3:**
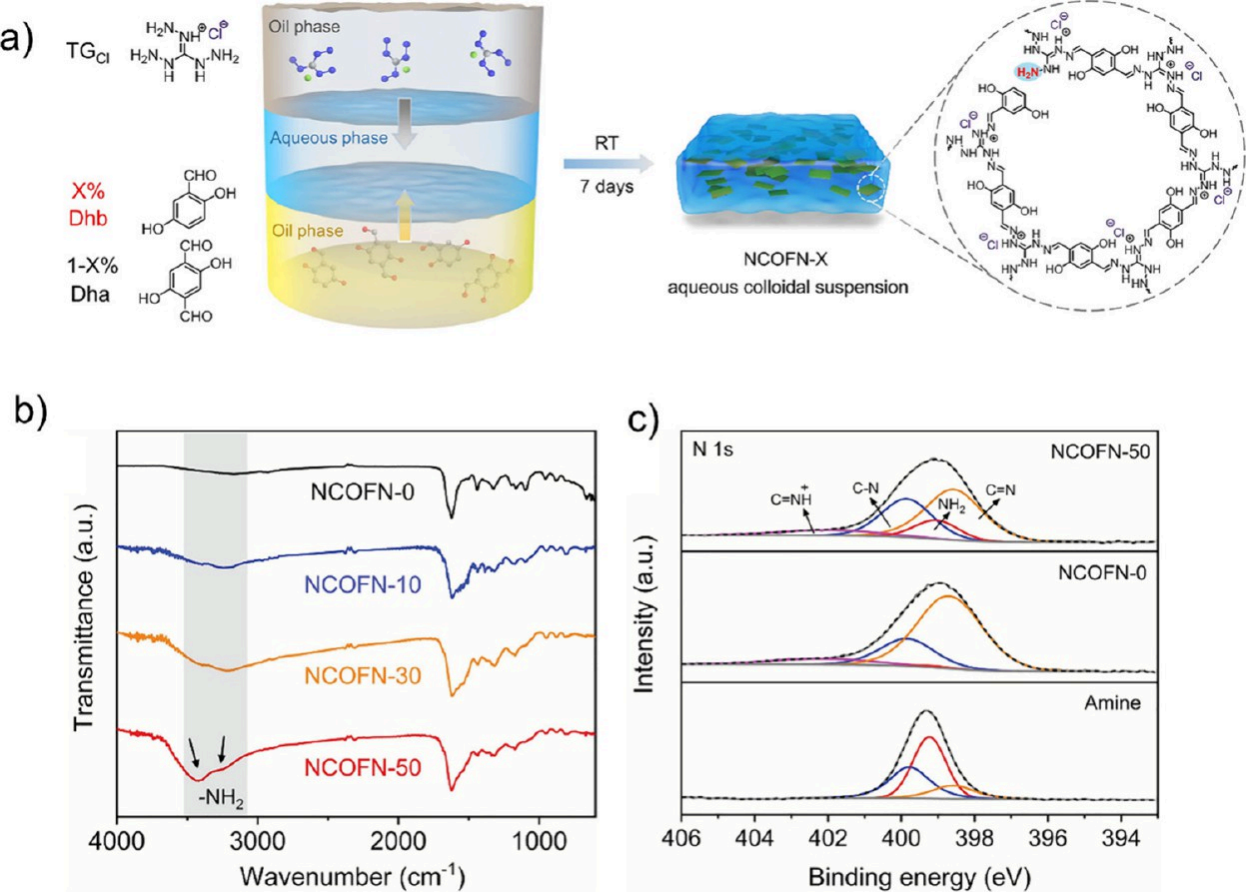
NCOFN-X design and synthesis. a) Schematics synthesis, b) FT-IR spectra, and c) XPS profiles of the nanosheets. Reproduced with permission from ref. [^35^]. Copyright 2020 John Wiley and Sons.

The presence of unreacted amino groups in the framework was confirmed by IR spectroscopy (Figure 3b).^35^ Notably, the intensity of IR bands corresponding to free amino groups increases with a higher proportion of monoaldehyde DHB, indicating more linker DHA vacancies. This observation suggests that the IR band intensity of amino groups can serve as a useful indicator of the defective COF structure with free amino groups.

FT-IR spectra were also used to check the controlled hydrolysis of some knots of the polyimide-based COF samples in alkaline solution. This process introduced carboxylate groups as defect sites within the material structure.^73^ Figure 4 illustrates the FT-IR spectra before and after KOH treatment. The characteristic peaks of intact imide rings (1780, 1720, and 1375 cm^–1^, corresponding to asymmetric and symmetric stretching of imide C=O and C–N–C moieties, respectively) show reduced intensity following KOH exposure, indicating the cleavage of cyclic imide linkages under alkaline conditions.

**Figure 4:**
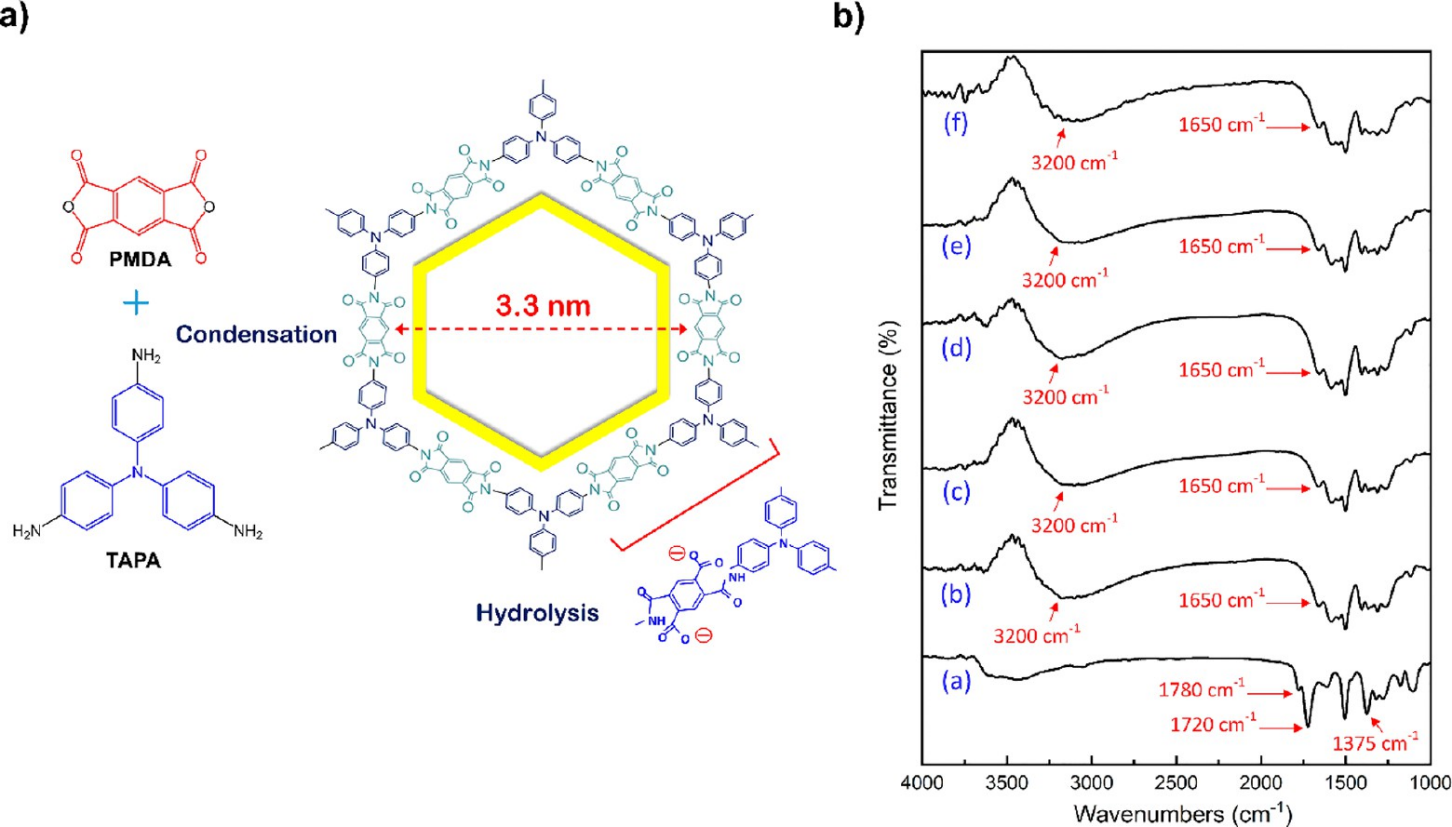
a) Schematic synthesis and b) FT-IR spectra of the imide-based COFs (a = primary COF, b-f refer respectively to the treated COF in 1, 2, 3, 4, and 5 M of KOH solution for 1 h). Reproduced with permission from ref. [^73^]. Copyright 2022 Elsevier.

In the meantime, new peaks at around 3200 cm^–1^ (attributed to N–H stretching bands) and 1650 cm^–1^ (assigned to C=O stretching vibrations) appeared in the IR spectra of defective COFs, corresponding to the cleaved imide rings. The FT-IR analysis, thus, confirms the successful generation of active carboxylate groups during the postsynthesis hydrolysis treatment. These newly formed functional groups serve as adsorption sites for the removal of organic dyes from the solution.

Li and co-workers investigated the difference between perfect and defective imine-based COFs (dCOF-NH_2_-Xs, where X = 20, 40, or 60 = [DHB]/[DHB + DHA (or DHTA)] × 100) by the appearance of new peaks of N–H stretching bands at 3300–3500 cm^–1^ in the FT-IR spectra.^36^ By controlling molar ratios of the monoaldehyde to the dialdehyde during condensation reaction with 1,3,5-tri-(4-aminophenyl)­benzene (TAPB), forming the dCOF, the missing-linker defects, and therefore, the content of unreacted −NH_2_ groups arising from the TAPB as defective sites could be precisely tuned (Figure 5).^78^

**Figure 5:**
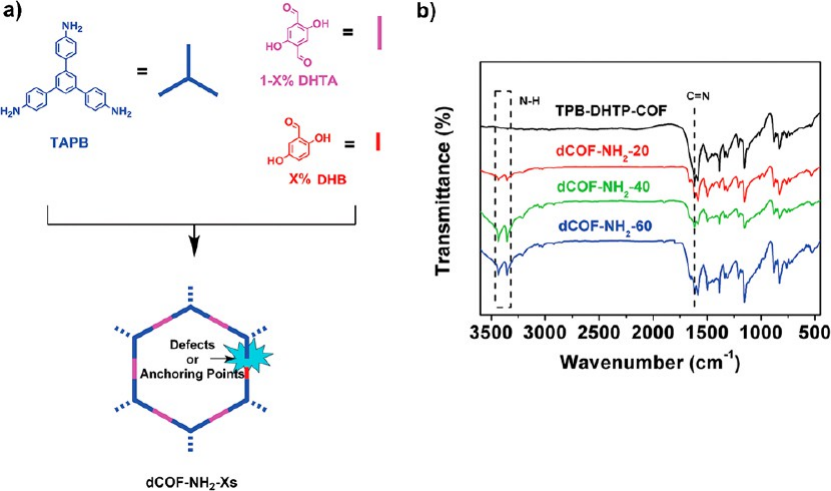
a) Synthesis and b) FT-IR spectra of the defective COFs. Reproduced with permission from ref. [^36^]. Copyright 2020 John Wiley & Sons.

There are also other relevant examples employing IR techniques in the literature.^34,36,46,47,51,74,78,81,90,92,93^

Diffuse reflectance mode infrared Fourier transform spectroscopy (DRIFT) is particularly useful for analyzing nontransparent, opaque samples. Unlike transmission mode IR spectroscopy, which requires transparent samples, DRIFT can provide information on the presence, quantification, and degree of functionalization of defects in powder samples without the need for sample preparation.^76,94–97^ As commented on earlier, the information provided by IR spectroscopy can be combined with that of the other characterization techniques to distinguish the presence of defective sites in COFs. IR spectroscopy is very suited to the use of probe molecules, particularly those that can be adsorbed from the gas phase, and can interact with the sites in the material and report on their density and strength. In MOFs, open metal sites refer to coordinatively unsaturated metal centers that can interact with guest molecules.^98^ Similar concepts can be applied to probing defects in COFs, even though they do not contain metal centers but organic functional groups. Typical probes like CO and pyridine can be used to titrate open binding sites or acid–base centers in the soft materials.^99–104^ In this case, IR spectroscopy monitors the changes in the spectra that occur upon adsorption and desorption of the probe under the controlled conditions of pressure and temperature. Similarly, the adsorption of reagents can result in the formation of reaction intermediates that can be detectable by IR spectroscopy. Following the evolution of these reaction intermediates with time, temperature, or upon ingress of other reagents can provide information on the reaction mechanism.

### 2.2 NMR

The introduction of accessible active sites can supply more opportunities for the development of new COF materials. Solution NMR spectroscopy of digested COFs has been broadly developed to detect and quantify defective sites in the COF structure and provide useful information on their surface functional groups.^36,74,75^ NMR spectroscopy can also be employed to probe the loading level of the postsynthetic defect functionalization. However, the exact identification of diverse kinds of vacancies in different proportions in a COF is still challenging. NMR spectroscopy and interpretation may need prior assumptions and considerations to correctly estimate the ratio of missing components.^74^ The materials are commonly digested in a mix of acid/base and deuterated solvent.

As an example, Bunck and co-workers used effectively a monomer truncation strategy to prepare a defective 3D boroxine-linked framework, namely COF-102-allyl, obtaining from the condensation tetrahedral tetrakis­(boronic acid) monomer with allyl-functionalized tris­(boronic acid) as a truncated monomer.^44^ The allyl (or defective sites) amount (∼22%) in COF-102-allyl was calculated from the ^1^H NMR spectra of the dissolved sample in CD_3_CN/D_2_O by the integration of the vinylic (A and B at 4.91 and 5.52 ppm) and alkylic (C at 3.41 ppm) protons with respect to the aromatic protons (Figure 6a, top). In addition, the COF-102-allyl functionalization with propanethiol (SPr) toward formation of COF-102-SPr via a typical thiol–ene reaction was checked by the ^1^H NMR spectrum as the three mentioned peaks related to the vinylic and allylic protons disappeared and the new peaks at 2.38, 1.42, and 0.84 ppm related to the aliphatic chains after the covalently attachment of SPr are observed (Figure 6a, bottom).

**Figure 6:**
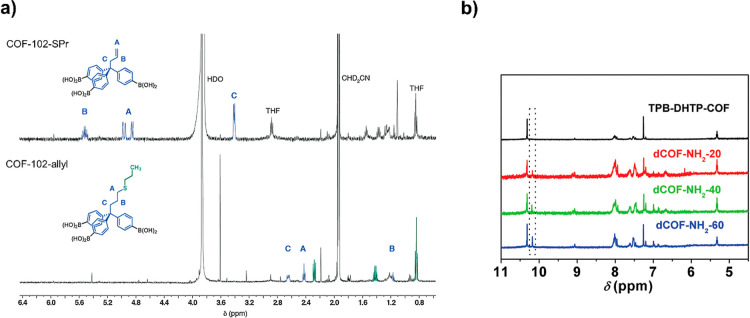
^1^H NMR spectra of a) dissolved COF-102-allyl and COF-102-SPr. Reproduced with permission from ref. [^44^]. Copyright 2013 Royal Society of Chemistry and b) dCOF-NH_2_-Xs. Reproduced with permission from ref. [^36^]. Copyright 2020 John Wiley & Sons.

In another work, ^1^H NMR spectra of the digested COFs (dCOF-NH_2_-Xs, X = 0, 20, 40, and 60) in HCl were considered to quantify the defect level by the integration of the monoaldehyde as the truncated monomer (10.18 ppm) and dialdehyde (10.32 ppm) protons, which were approximately the same as the feed ratios (Figure 6b).^36^

In another work, two types of structural vacancies (aldehyde- or amine-based components, Cu_3_(PyCA)_3_ or PA vacancies; PyCA = pyrazolate-4-carboxaldehyde, PA = *p*-phenylenediamine) in digested FDM-71 (FDM = Fudan materials, a MOF/COF hybrid) in diethyldithiocarbamate ((C_2_H_5_)_2_NCSSNa) in DMSO-*d*_6_/D_2_O were fruitfully distinguished and quantified by using solution ^1^H NMR analysis (Figure 7).^74^ FDM-71 is a 2D porous structure with Cu_3_(NN)_3_ nodes and the *in situ* created *N*,*N*′-bis­[(pyrazol-4-yl)­methylene]-benzene-1,4-diamine (BPBD) organic connections. When PA missing arises, two PyCA are missed from the network. Deficiency of a Cu_3_(PyCA)_3_ is also complemented with the removal of three *N*-[(pyrazol-4-yl)­methylene]-benzene-1,4-diamine (PBD). Accordingly, based on the NMR analysis and the correct postulation, the percentages of PA (∼24%) and Cu_3_(PyCA)_3_ (∼2.8%) deficiencies in FDM-71 are obtained and quantified from the molar ratio of BPBD:PyCA:PBD in the solution. It should be noted that the (C_2_H_5_)_2_NCSSNa disconnects selectively the Cu-pyrazolate coordination against the imine covalent bonds.^74^

**Figure 7:**
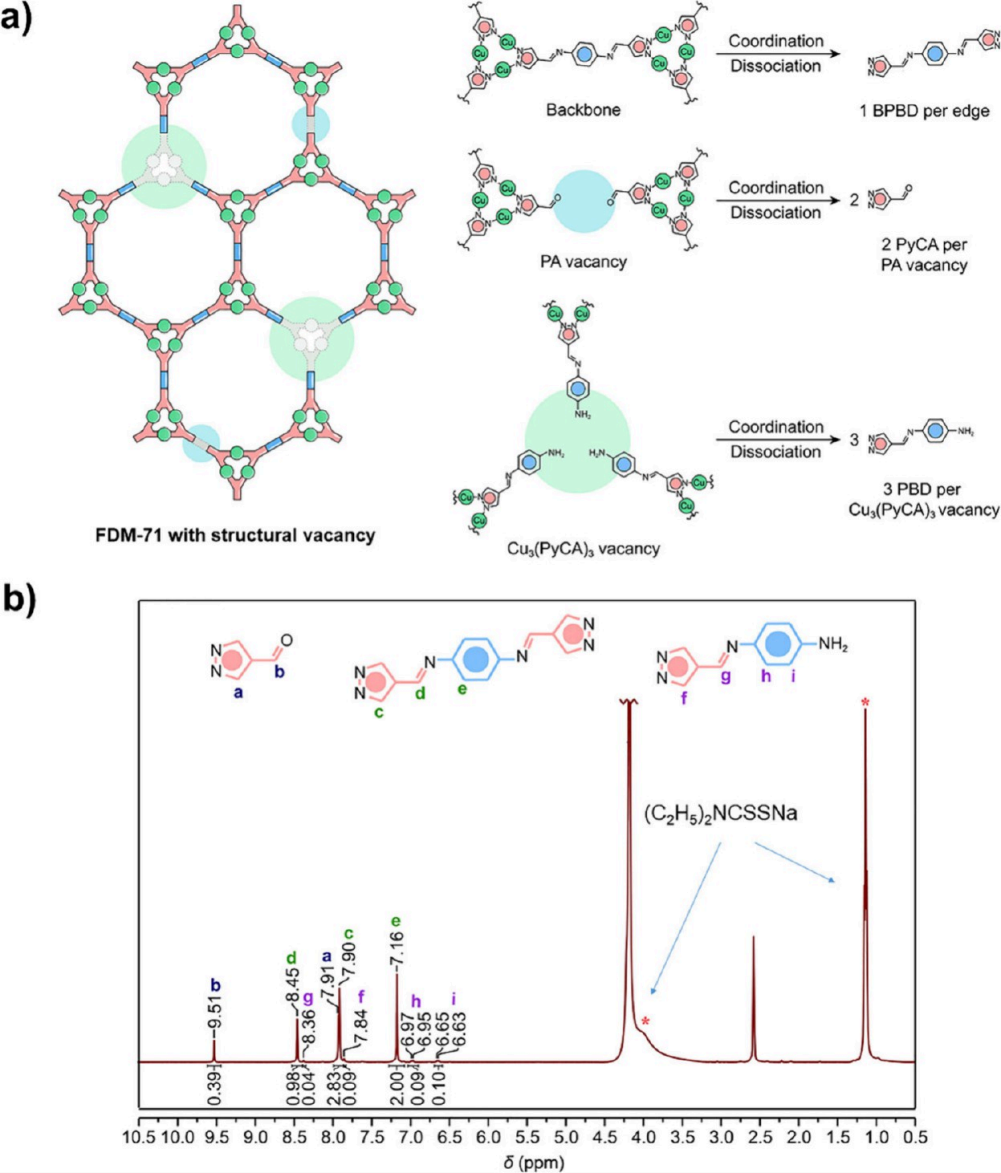
a) Defective FDM-71 and b) its ^1^H NMR spectrum (BPBD:PBD:PyCA = ∼ 1:0.08:0.69). Reproduced with permission from ref. [^74^]. Copyright 2021 John Wiley & Sons.

Solid-state NMR (ss-NMR) spectroscopy, particularly ^13^C ss-NMR, is also an influential atomic-level tool to investigate the chemical structure and layer stacking within COF layres.^36,78,105^ However, generally, its low sensitivity and overlapping signal patterns limit its application.^35^ Dichtel group used cross-polarization and magic angle spinning (CP-MAS) ^13^C ss-NMR to show incomplete condensation and free aldehyde groups in an imine-linked hexagonal 2D COF.^75^ This happened when bifunctional monomers (biphenyldicarbaldehyde (BDA) and terephthalaldehyde (PDA)) with different lengths and 25–75 mol % of BDA were incorporated into the COF.

In another work, a defective CTF (CTF-SD_2_) bearing cyano defects and B dopants was prepared through the calcination of a mixture of primary CTF and NaBH_4_ in the absence of air.^93^ Obviously, the ^13^C MAS NMR spectrum confirmed the structure by the appearance of two new peaks at 112.6 and 171.9 ppm, related to the −CN groups (**d**) and C atom in the C–B pieces (**e**), as compared with that of the original CTF (Figure 8).

**Figure 8:**
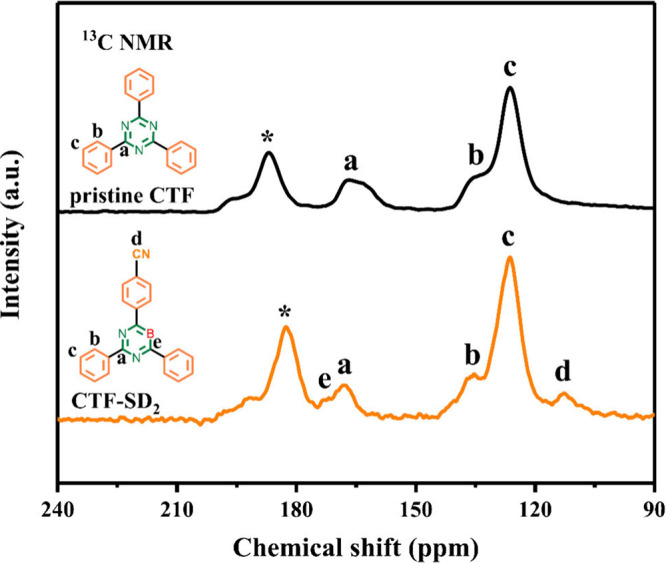
^13^C ss-NMR spectra of the original and defective CTFs. Reproduced with permission from ref. [^93^]. Copyright 2022 American Chemical Society.

Applying in-depth heteronuclear NMR and combining it with elemental analysis can further allow for precise determination and quantification of defect degrees in the frameworks.^35,36,93^

### 2.3 TGA

This is a common tool to indirectly detect the existence of defects in COFs. The obvious difference in weight loss between the ideal and defective COFs upon thermal treatment can be used to assess differences between them. Similarly to the studies on defective MOFs by TGA in which decomposition of the organic moiety occurs first and there is a final residue due to the metal.^106,107^ TGA measurements can be used to estimate the amounts of missing linker defects in COFs and their functionalization. Such types of TGA are routinely performed to distinguish defects in COFs.^36^ Additionally, TGA profiles can reveal increased COF stability due to variations in the stacking modes of 2D COFs.^56^ A notable example of TGA application in COF defect analysis was reported by Han and colleagues in 2020.^36^ They used TGA to investigate the presence and functionalization of defects in the COFs. By comparing the three-stage weight loss plateaus of an ideal COF (TPB-DHTP-COF), a defective COF (dCOFNH_2_-60), and a functionalized sample (dCOF-ImBr-60, where ImBr is a brominated imidazole derived from bromoimidazole carbaldehyde), it was possible to discern missing-linker defects and their functionalization (Figure 9). The lower observed weight loss in defective COFs compared to ideal ones indicates the presence of linker vacancies in the framework. Derivatization of defective COFs results in changes to the TGA curve, corresponding to weight loss from newly introduced units and/or cleaved connections.

**Figure 9:**
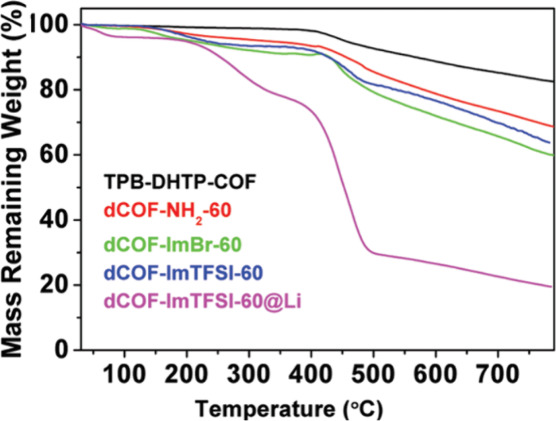
TGA profiles of TPB-DHTP-COF, dCOF-NH_2_-60, dCOF-ImBr-60, dCOF-ImTFSI-60, and dCOFImTFSI-60@Li. Reproduced with permission from ref. [^36^]. Copyright 2020 John Wiley and Sons.

Despite its utility, TGA has certain limitations in the detection of defects. The technique cannot provide detailed structural information to accurately identify the nature of the various defect types or their precise locations within the framework. As such, although TGA is a valuable tool for detecting defects in COFs, it should be used in conjunction with other analytical methods for comprehensive defect characterization.

While TGA can be a useful technique for probing defects in COFs, it is important to consider carefully those structural factors that may lead to artifacts in the analysis, forming disordered phases before degrading to volatile byproducts. As discussed by Xu et al.,^76^ factors such as size of the crystalline COF lattice, pore functionalization, structural distortions, framework flexibility, interlayer interactions, and thermal expansion can affect TGA results. These parameters should be carefully considered when TGA data are interpreted for defect analysis in COFs.

### 2.4 Nitrogen Sorption

Nitrogen adsorption–desorption isotherms provide information on the specific surface area of a material by determining the point of monolayer coverage and also the corresponding pore size distribution by measuring the point of capillary filling of the void spaces. Thus, gas adsorption is another tool to gain information on the presence of defects in porous materials.^108^ The existence and concentration of defects in the COFs would certainly cause deviations in the surface area, pore volume, and pore size distribution of the sample compared to pristine frameworks.^36,43^ In addition, surface area and pore size distribution measurements can be employed to assess the suitability of structural models to represent the real COF sample, as for instance to distinguish between models corresponding to the eclipsed and staggered packing structure in COFs can be used to probe the packing modes in COFs.^74,109^

Capillary condensation is a phenomenon where gas molecules condense in small pores at pressures below the saturation vapor pressure of the bulk liquid. This process can lead to hysteresis patterns in nitrogen adsorption–desorption isotherms of the COFs. These hysteresis patterns can serve as an effective technique for probing mesoporous defects in COFs. The shape and width of the hysteresis loop can provide information about pore size distribution and the presence of defects that create mesopores within the COF structure.^71,77^

In one interesting example aimed at finding a correlation among defects, surface area, and pore size distributions, Han et al. measured the Brunauer–Emmett–Teller (BET) surface areas of COF samples containing different numbers of defect sites (dCOF-NH_2_-Xs, X = 20, 40, and 60 containing -NH_2_ groups) and dCOF-CHO-Xs (X = 20, 40, and 60 containing −CHO groups).^36^ They found that the larger proportion of missing-linker defects resulted in a smaller BET surface area of the sample. In contrast, the pore size increased as the density of defect increased as calculated from the nitrogen adsorption isotherms applying nonlocal density functional theory method.

In the study reported by Jiang and co-workers,^35^ an increase in the number of missing linkers and the density of amino-functionality resulted in an increase in pore volume of the defective NCOFN as compared with the defect-free NCOFN.

Based on the comparison of the simulation data with the experimental surface area, pore volume and pore width values, Li et al. concluded that the FDM-71 resulting of the condensation of PA and Cu_3_(PyCA)_3_ having the structural defects is packed in an eclipsed mode with mesoporous hexagonal channels (see Figure 10 and Table 2) rather than staggered microspores form.^74^

**Figure 10:**
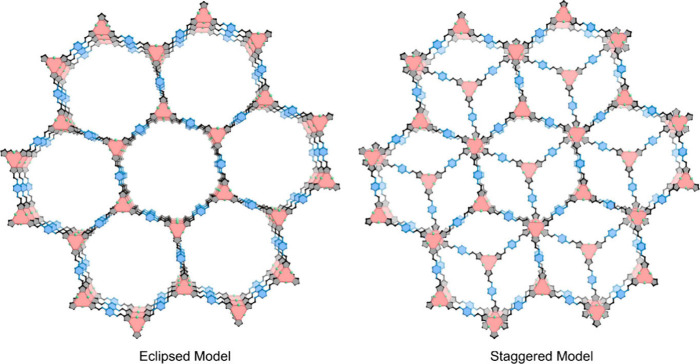
Simulated FDM-71 structures with eclipsed or staggered packing. PA component is blue; the PyCA component is black; the Cu_3_(NN)_3_ nodes are light red; Cu, green. H atoms are omitted for simplicity. Reproduced with permission from ref. [^74^]. Copyright 2021 John Wiley and Sons.

**Table 2:** Theoretical and Experimental Pore Characteristics of FDM-71 ^74^

	BET surface area (m^2^ g^–1^)	Pore volume (cm^3^ g^–1^)	Pore width (Å)
Eclipsed model	1547^a^	1.200^a^	26.2^a^
Staggered model	1849^a^	0.899^a^	8.8^a^
FDM-71	1135^b^	1.166^b^	25.8^b^

a Simulated data, the calculation is based on defect-free FDM-71 structure with all Cu sites in reduced form.

b Experimental data.

### 2.5 Acid–Base Titration

Acid–base titration can provide valuable quantitative information about the density of acidic or basic defect sites in COFs.^78,110,111^ This technique can be particularly useful for COFs with intentionally introduced acidic or basic functional groups.^112^ However, it is important to note that this method may not be suitable for all types of defects and may be limited by the accessibility of the titrating probe to the defect sites. Additionally, the choice of solvent and titrating agent can significantly impact the results, as they may affect the COF structure or interact differently with various types of defects. An example reported in 2018,^78^ in which the concentration of acidic protons (−COOH groups) in COF-1 determined employing the titration system. For this purpose, the COF was placed in an ion-exchange agent and filtered off, which was later acidified by HCl and titrated by using a solution of NaOH at room temperature to complete the acid–base neutralization. In addition, Toluidine Blue O (TBO) colorimetric assay^113,114^ has been also reported for the simple quantification of −COOH groups in the defective COF-1.^78^

### 2.6 XRD

XRD techniques, including SC-XRD and PXRD, are crucial tools for the structural characterization of COFs. However, their effectiveness in detecting and analyzing defects varies.^115,116^ SC-XRD is a powerful method for determining the precise structure of COFs, but it often fails to detect defects directly.^60,117^ In addition, too small sizes or low crystal qualities are not suitable for SC-XRD, and 3D electron diffraction methods are also needed for the structure determination.^115^ The crystal phase is qualitatively determined by comparing the relative peak intensities to highly crystalline samples. While various strategies have been developed to prepare suitable samples for SC-XRD, examples of single crystals for defective COFs are rare, limiting discussion of this technique in defect analysis.

PXRD, on the other hand, is more commonly used for structural characterization of polycrystalline materials.^118,119^ It can provide information on crystallinity, phase purity, and stacking modes by comparing experimental patterns with simulated ones from SC-XRD data or computational models.^56,74,80,120,121^ Well-regulated stacking modes of 2D COFs can meaningfully tune their properties and functionalities, but their synthetic procedures are still challenging.^56^ PXRD patterns are also used to determine interplanar distances using Bragg law.^79^

The research done by Han et al. shows that the controlled defects in COFs does not change significantly its primary crystallinity.^36^ Similarly, based on the comparison of their XRD patterns, Jiang et al. indicated that the crystalline network of the defect-engineered NCOFN nanosheets was identical to that of defect-free NCOFN.^35^

While controlled defects in COFs may not always lead to significant changes in the XRD pattern, a recent study has shown that certain types of defects can affect the crystallinity. For example, Zhao et al.^43^ demonstrated a correlation between defects and changes in crystallinity for some COF systems, such as defective COF-HNU30-X (*X* = 5, 10 and 20, where *X* is calculated from [DHB]/[DHB + DHA (or DHTA)] × 100,). The impact of defects (missing-linker) on XRD patterns can depend on the concentration of the monoaldehyde as terminating units (DHB) and exactly control the molar ratios of building blocks. Comparatively, the crystallinity and reflections were improved from defect-free COF-HNU30-0 to COF-HNU30-5 and COFHNU30-10, and then decreased to COF-HNU30-20 and COF-HNU30-30 with a higher concentration of defects. Therefore, the careful analysis of XRD data, potentially combined with other characterization techniques, is crucial for a comprehensive understanding of defects in COFs.

Bojdys et al.^79^ showed that a triazine-based COF, covalent triazine frameworks CTF-2, with an interplanar stacking distance of 3.36 Å, contained eclipsed ordering stacking domains interspersed with other regions of staggered or arbitrary packing modes or other possible defects such as screw dislocation. This conclusion was reached by comparing the calculated patterns for eclipsed and staggered packing with the experimental PXRD pattern of CFT-2 (Figure 11a).

**Figure 11:**
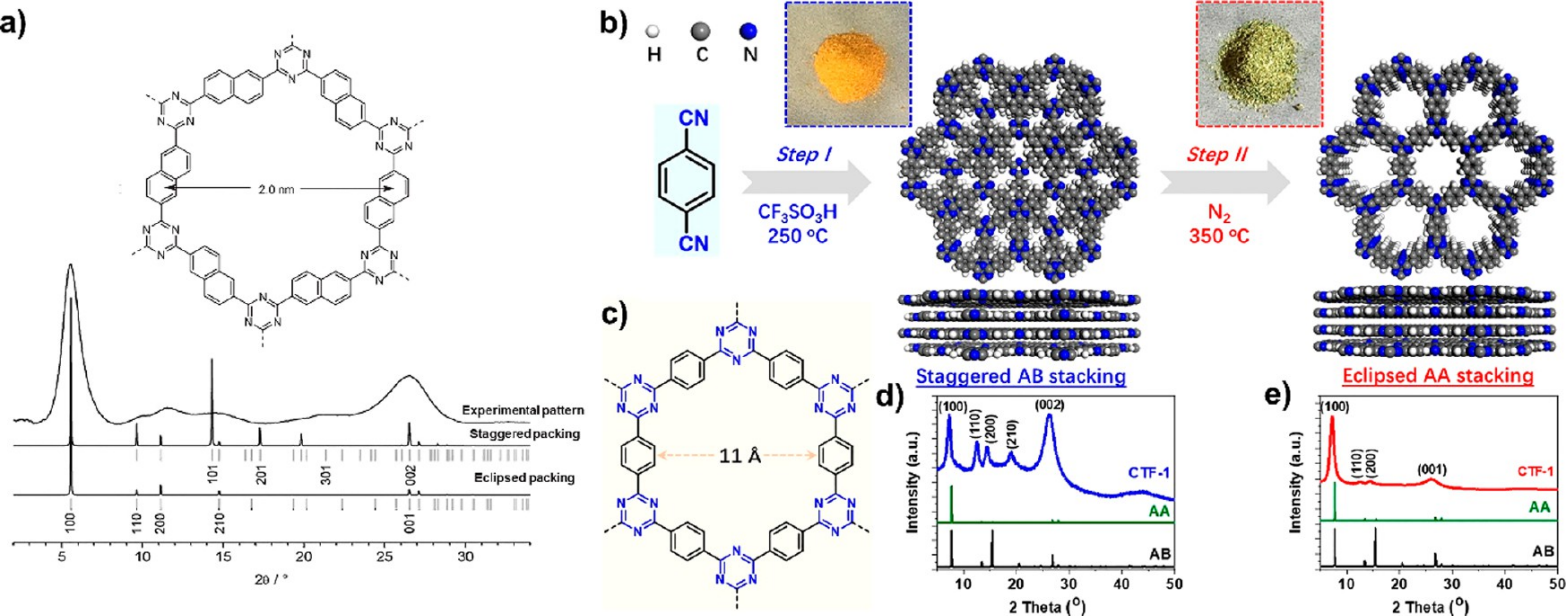
a) Structure and PXRD patterns (simulated and experimental) of CFT-2 Reproduced with permission from ref. [^79^]. Copyright 2010 John Wiley and Sons. b) Synthetic procedures toward formation of CTF-1 with AB and AA stacking order, c) unit cell in CTF-1, and d, e) simulated and experimental PXRD patterns of the CTF-1 framework with different stacking modes. Reproduced with permission from ref. [^80^]. Copyright 2020 American Chemical Society.

Yang et al. showed that CTF-1 with a staggered AB stacking mode achieved by using trifluoroacetic acid (TFA) is successfully transformed to eclipsed AA stacking mode under thermal and inert atmosphere condition as marked by PXRD (Figure 11b-e).^80^

Wang et al.^56^ reported an interesting procedure to switch the layer stacking of imide-linkage 2D COFs (NKCOF-11, NKCOF stands for Nankai covalent organic framework) by changing the synthetic processes. Based on the PXRD data, there were two stacking modes (AA or ABC order) for NKCOF-11 (Figure 12). An interlayer distance of ABC mode was calculated 3.08 Å, which was lower than that of the AA mode (3.81 Å). The COFs with different modes showed different gas adsorption performance.

**Figure 12:**
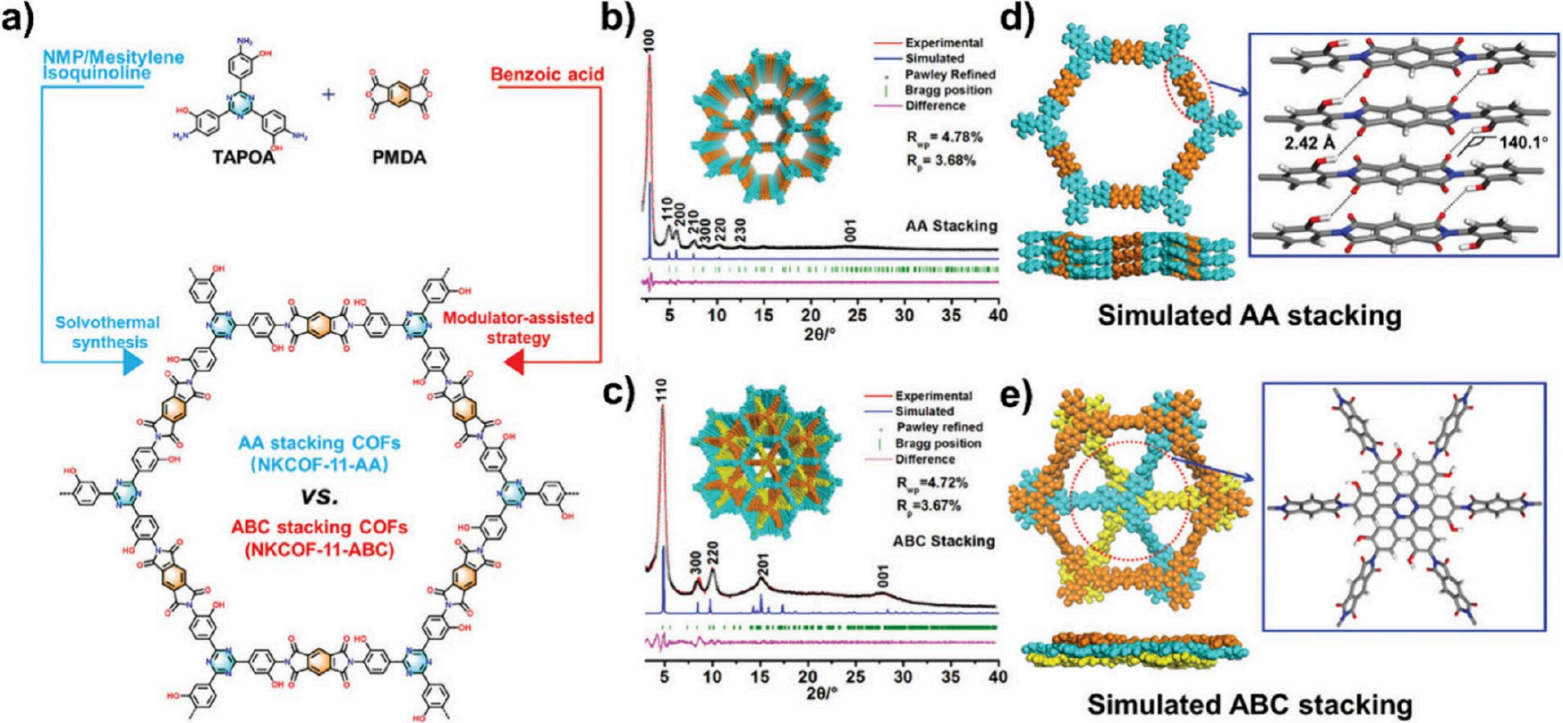
a) Schematic synthesis of the NKOF-11. b, c) PXRD patterns of the framework with AA and ABC stacking modes; experimental (red), Pawley-refined (black), and the simulated patterns (blue). d,e) Top and side views of the framework. Reproduced with permission from ref. [^56^]. Copyright 2023 John Wiley and Sons.

In addition to X-ray diffraction, X-ray absorption fine structure (XAFS) spectroscopy, including extended X-ray absorption fine structure (EXAFS), is another common X-ray technique based on the use of very bright radiation beams. Due to its high intensity, the resulting EXAFS has a better resolution and can report on minor variation and local structure of the solid, measuring the coordination numbers (N), bond distance (R), and Debye–Waller factor (s2). However, EXAFS is rarely used for determining the binding environment and size of metal species located into the defective COFs,^122^ which act as a support for single-atom catalysts.^123^

### 2.7 XPS

XPS analysis is routinely used to analyze elemental compositions, valence states, chemical bonds and coordination sphere of the elements present on the surface or outermost layers of materials.^74,93^ Interestingly, XPS analysis was used to calculate and quantify the defect level in the NCOFN nanosheets (see above Figure 3c).^35^ Accordingly, the amount of free amino groups or defect sites were obtained 9.7%, 27.7%, and 38.4%, respectively, for NCOFN-10, NCOFN-30, and NCOFN-50. The results were in accordance with the −NH_2_ defect content of NCOFN nanosheets estimated from the elemental analysis. In another work, XPS analysis was used to determine the introduction of cyano groups into CTFs as the result of the cleavage of the triazine heterocycles with NaBH_4_, offering defective CTFs (CTF-SDs).^93^ In addition, the successful incorporation of various organic compounds into the defective sites of COFs via a covalent coupling strategy has been further evaluated by XPS.^36,81^

Since the penetration depth of the soft X-rays used in XPS is very shallow (1–2 nm), this technique can analyze only the external layers of the particles. In the case of COFs, where no surface oxidation or hydration by ambient atmosphere is expected, the composition of this external surface should match that of the bulk material. Nevertheless, since defects can be preferentially located on the external surfaces, XPS analysis can provide very useful information about the surface properties and density of defects. In fact, missing comonomers and the occurrence of dangling bonds on the surface could be addressed by XPS. Combining XPS analysis with fast-ion bombardment that cleans the surface and detaches some of the outermost layers allows mapping the elemental composition as a function of the penetration depths up to 100 nm. Variations in the relative atomic ratio upon penetration inside the material, even in thin layers, can also report on defect location.

While surface probing techniques primarily analyze the external layers of COF particles, in some cases, this surface analysis may correspond well to the composition and structure of the whole material. However, this assumption may not hold true for all samples, especially those containing metals that can oxidize upon exposure to ambient conditions. It is important to consider how surface defects such as dangling bonds can influence defect quantification. XPS depth-profiling techniques can provide additional insights by analyzing the composition of the material at various depths, potentially revealing differences between surface and bulk defects.

### 2.8 DLS

DLS also known as photon correlation spectroscopy or quasi-elastic light scattering is one of the most important tools for measuring the size distribution (up to the nm region), polydispersity index, and zeta potential of defective-COF particles in suspension.^74,124^

In one study reported by Zhen et al., the hydrodynamic size distribution obtained from DLS of the water dispersions was used to probe the different defect degree in dCOF-NH_2_-Xs.^36^ The results showed that as the defect concentration increased, the particle size decreased from ∼690 nm (X = 0) to ∼335 nm (X = 60) (polydispersity index: TPB-DHTACOF = ∼ 0.638; dCOF-NH_2_-20 = ∼ 0.687; dCOF-NH_2_-40 = ∼ 0.857; dCOF-NH_2_-60 = ∼ 0.729) (Figure 13). This observation is in line with the general rule that smaller particles tend to have more surface defects. In this case, the increased defect concentration due to more end-capping units employed likely limits polymer growth, resulting in smaller particles. This highlights the complex relationship between defect engineering and particle size in COFs, which can be influenced by factors such as reaction kinetics and growth mechanisms.

**Figure 13:**
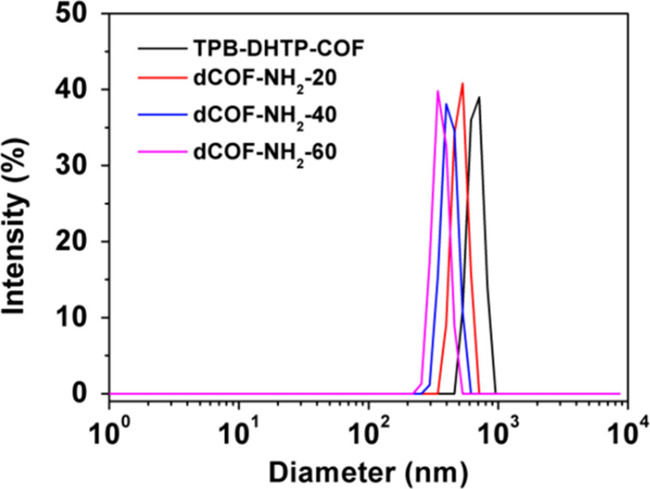
Average size of dCOF-NH_2_-Xs measured by DLS (water solutions). Reproduced with permission from ref. [^36^]. Copyright 2020 John Wiley and Sons.

In another study, particle size, polydispersity index, and zeta potential measurements from DLS further supported the successful synthesis of defective COF (**1**), and its functionalization with a Porphyrin (COF-Por (**2**)) and metal complexation with a V=O moiety VONc@COF-Por (**3**) (Figure 14).^45^

**Figure 14:**
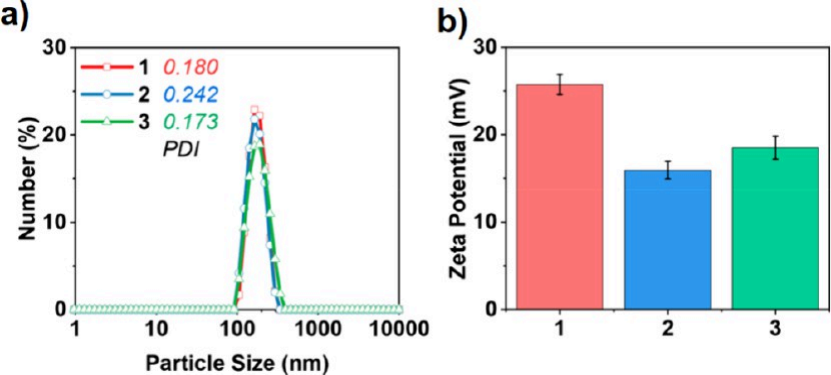
a) DLS size profiles and polydispersity index (PDI) of **1**, **2**, and **3** and (b) their zeta potentials in phosphate-buffered saline (pH 6.5). Reproduced with permission from ref. [^45^]. Copyright 2019 American Chemical Society.

The zeta potential of defective FDM-71 particles in aqueous media was also found to be 17.4 ± 0.3 mV by DLS, displaying its good dispersity in H_2_O, and its hydrodynamic size distribution was found about 122 nm.^74^

### 2.9 Electron Microscopy Techniques

SEM, TEM, and STEM are also often employed to detect the degree of defects introduced in the as-prepared defective COFs.^36^ TEM and STEM are among the useful techniques for checking long-range ordered arrangement at the atomic level, while STM and SEM can achieve the surface structure information.^125^ Comparing defective COFs to nondefective COFs can be useful to understand the impact of defects on morphology and particle size. However, it is important to note that advanced synthetic techniques for generating defects may alter reaction kinetics and growth factors, leading as a consequence to size differences unrelated to the defects themselves. Additionally, factors such as secondary nucleation assisted Ostwald ripening^126,127^ and excess reactive functionalities (such as amine and aldehyde in imine-linked COFs arising from missing knots and linker defects) can influence also morphological outcomes. These aspects should be carefully considered when interpreting morphological differences between defective and nondefective COFs.^43^ In one of the recent examples, SEM and TEM images have shown highly analogous morphologies between the nondefective and defective COFs.^36^ In addition, the average *d* spacing value of obtained from XRD analysis can be checked by HRTEM.^35^

From a defect engineering perspective, some reactions employed in the COF synthesis or modification are irreversible. These irreversible reactions can be strategically used to either enhance or intentionally disrupt crystallinity and porosity, depending on the desired application and properties of the COF. The structure of most soft materials, including COFs, is vulnerable to high-energy electron beams, making it challenging to use HRTEM for observing atomic-level defects in COF structures and arrangements.^52,82–84^ To address this issue, postsynthetic locking (PSL) strategies have been developed. PSL, a subclass of PSM, is useful for creating irreversible bonds and electron beam-stable COFs. This allows for more in-depth imaging and analysis of structural defects and disorder while expanding framework applications.^51,128,129^ Overall, the PSL helps to minimize the inherent limitations associated with reversible bonds in the COFs.

Grain sizes and grain boundaries are significant types of defects in COFs that can considerably impact their properties.^54^ These defects can be probed using techniques such as TEM and STEM. TEM can provide direct visualization of grain boundaries, while STEM can offer elemental mapping to identify compositional variations at grain interfaces. Also, the impact of grain boundaries on thermal stability can be indirectly probed by using TGA, as these defects may affect the overall decomposition behavior of the COF.

For instance, Haase et al. used a topochemical PSL, converting an imine-based CTF (TTI-COF) to the robust thiazole bonds (−S–C=N-, TTT-COF­(triphenyl triazine thiazole COF)), to monitor the formed edge dislocations/linker vacancies as defects in the framework by TEM (Figure 15).^51^ Interestingly, they designated defects that are intrinsically formed during reversible imine condensation.

**Figure 15:**
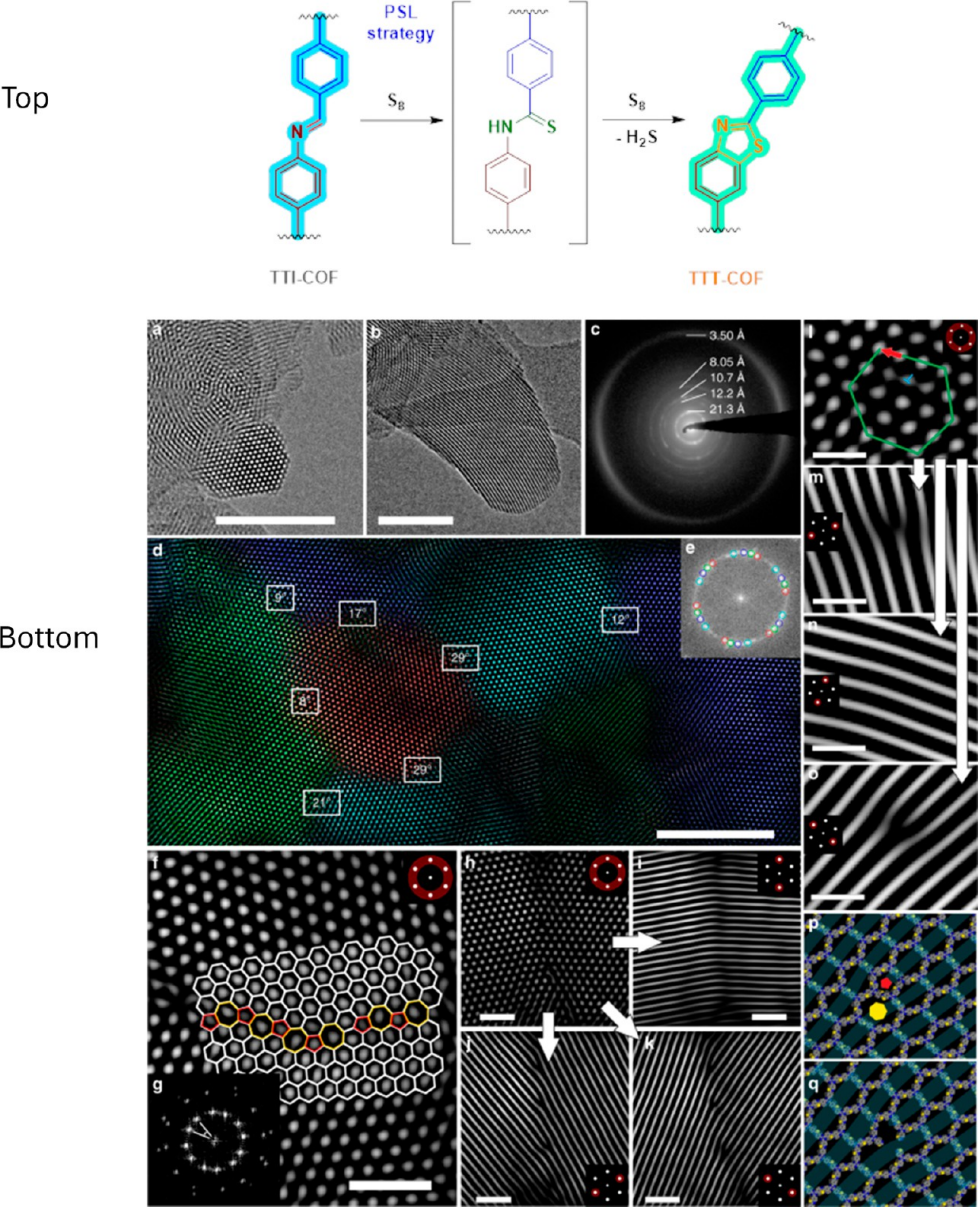
top) Topochemical conversion of imine COF to thiazole-linked polymer (TTI-COF to TTT-COF) and bottom) TEM characterization of TTT-COF. a,b) Hexagonal channels. c) Selected area electron diffraction (SAED). d) Intergrown grains with color overlay (e) showing distinct crystallites. f) High-angle grain boundarieswith 5- and 7-membered rings in comparison to the normal 6 membered rings. g) Fast Fourier transform (FFT) of (f). h-k) Low-angle grain boundaries. l-o) Edge dislocation analysis. p, q) Simulated edge dislocation via screw dislocation along the pentagonal (red pentagon) and the heptagonal channel (yellow heptagon) and missing-linker defects. Reproduced from ref. 51. Available under a CC-BY license. Copyright 2018 Haase, F. *et al*.

Later, Dichtel et al.,^52^ have developed a fast automated postprocessing Fourier mapping approach for deriving the information from HRTEM of beam-sensitive boronate ester-linked COFs. This technique provided rapid and valuable information beyond traditional imaging about defects and edge dislocations present in the frameworks, coming here from broken B–O bonds formed during polymerization.

### 2.10 STM

This is a scanning probe technique based on the tunnel effect in quantum mechanics for imaging surfaces and self-assembled organic molecules with atomic resolution.^87,130–133^ However, due to the occurrence of the often fast kinetics of the molecular self-assembly, most of the works have focused only on the morphology and properties of the resulting structures rather than their formation mechanism.^134,135^ Unlike SEM and TEM, STM does not drive electrons to a sample, and its resolution does not depend on the wavelength of the electron. Ourdjini et al., studied the effect of the metal nature (Ag(111) and Ag(100), Au(111), Cu(111)) as template on the synthesis of boronate COFs by using STM, presenting their characteristic arrangements and defects in Figure 16.^87^ Clearly, the images showed that Ag(111) and Ag(100) surfaces are better than the others for perfect COF synthesis.

**Figure 16:**
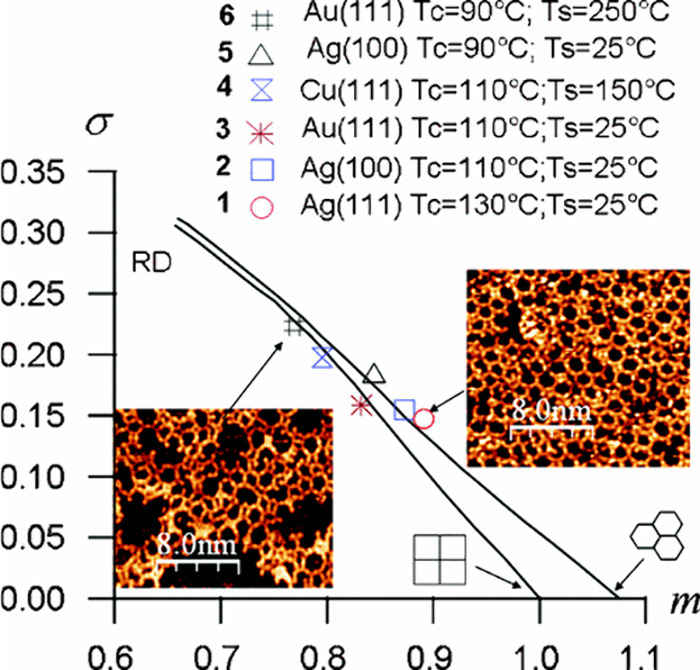
(m, σ) diagram for 1,4-benzenediboronic acid dimer deposited on the metal surfaces, derived from minimal spanning tree (MST) analysis. Data points represent samples heated at 200–300 °C, except the triangular point. Relative positions between perfect hexagonal and random arrangements indicate 2D COF polymer quality and/or defects. Reproduced with permission from ref. [^87^]. Copyright 2011 American Physical Society.

In a surface synthesis, Feyter and co-workers have successfully demonstrated that by applying an oriented electric field in a STM, the condensation of boronic acid trimers can be controlled facilely at the liquid/solid interface. The phase transition between self-assembled molecular pathways and formation/arrangements of COFs is also controlled by adjusting the polarity of the bias potential.^136^ In a similar work, the supramolecular self-assembly of COF-1 containing the grain/loop boundary defects were also evaluated by STM technique (Figure 17).^64^

**Figure 17:**
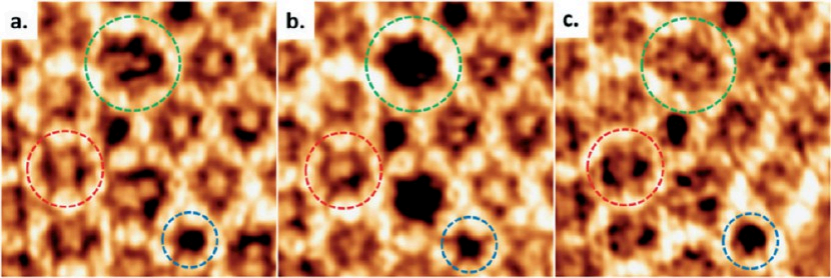
Sequential STM images (a-c) of COF-1, highlighting grain boundary defects containing pentagonal (blue), hexagonal (red), and heptagonal (green) rings. Reproduced from ref. 64. Available under a CC BY-NC license. Copyright 2017 Cui, D. *et al*.

Overall, despite the successful achievements outlined above, this technique is capable of controlling the polymerization around the STM tip. To solve this issue, an electrochemical STM (EC-STM) can be used.^137^

### 2.11 Computational Calculations

Theoretical calculations are also urgently needed to attain data, which are difficult to attain from experimental results. Combining experimental results and theoretical calculations are useful to establish structural features properly.^50,138–140^ Density functional theory (DFT) calculations can be used to model the structure and energetics of COFs, including the presence of defects.^39,63^ By the construction of computational models of COFs with different types of defects (e.g., missing linkers, substitutional defects, or structural distortions), the energetic stability and electronic properties of these defective structures can be evaluated. Molecular dynamics (MD) simulation is used increasingly to simulate properties of molecular structures of different morphologies and explore dynamics detailing on atomic scale through the calculation of interaction energy, driving forces, and binding energy of reversible reactions.^53^ MD simulations combined with a polymerization model and an Arrhenius two-state model have been employed to understand the evolution of reversible reactions as well as to elucidate the nucleation and growth dynamics in proving the defect healing mechanisms in 2D COFs,^88^ and thereby, targeting higher-quality COFs.^88,141,142^ For instance, Zhu et al. have explored mechanisms of defect correction in COFs toward the production of single crystals.^88^ Concisely, the small *E*_bind_ was essentially responsible for highly reversible reactions assisting the creation of single crystals by preventing nucleation and simultaneously slowing the growth for effective defect correction through avoiding the defects inside crystals.^88^ On the other hand, the formation of products with polycrystals and crystallinities was formed at moderate *E*_bind_ value, which increased the rate of both nucleation and growth. A higher growth rate incorporates defects inside crystals and results in the reduction of the crystal sizes. Furthermore, at very large *E*_bind_ value, the amorphous structures with defective polygons are preferred because of ineffective defect correction. In another work, by using MD simulations, they found pentagons and heptagons as structural defects, respectively, for combination [C_4_ + C_4_] and [C_4_ + C_2_] and for [C_3_ + C_3_] and [C_3_ + C_2_], during the growth of hexagonal frameworks (Figure 18a-f).^63^ With the help of density DFT calculations, they also found that the imine condensation reaction of TPA-COF and the boronate esterification reaction of COF-5 both experience two transition states with the structures shown in Figure 18g.

**Figure 18:**
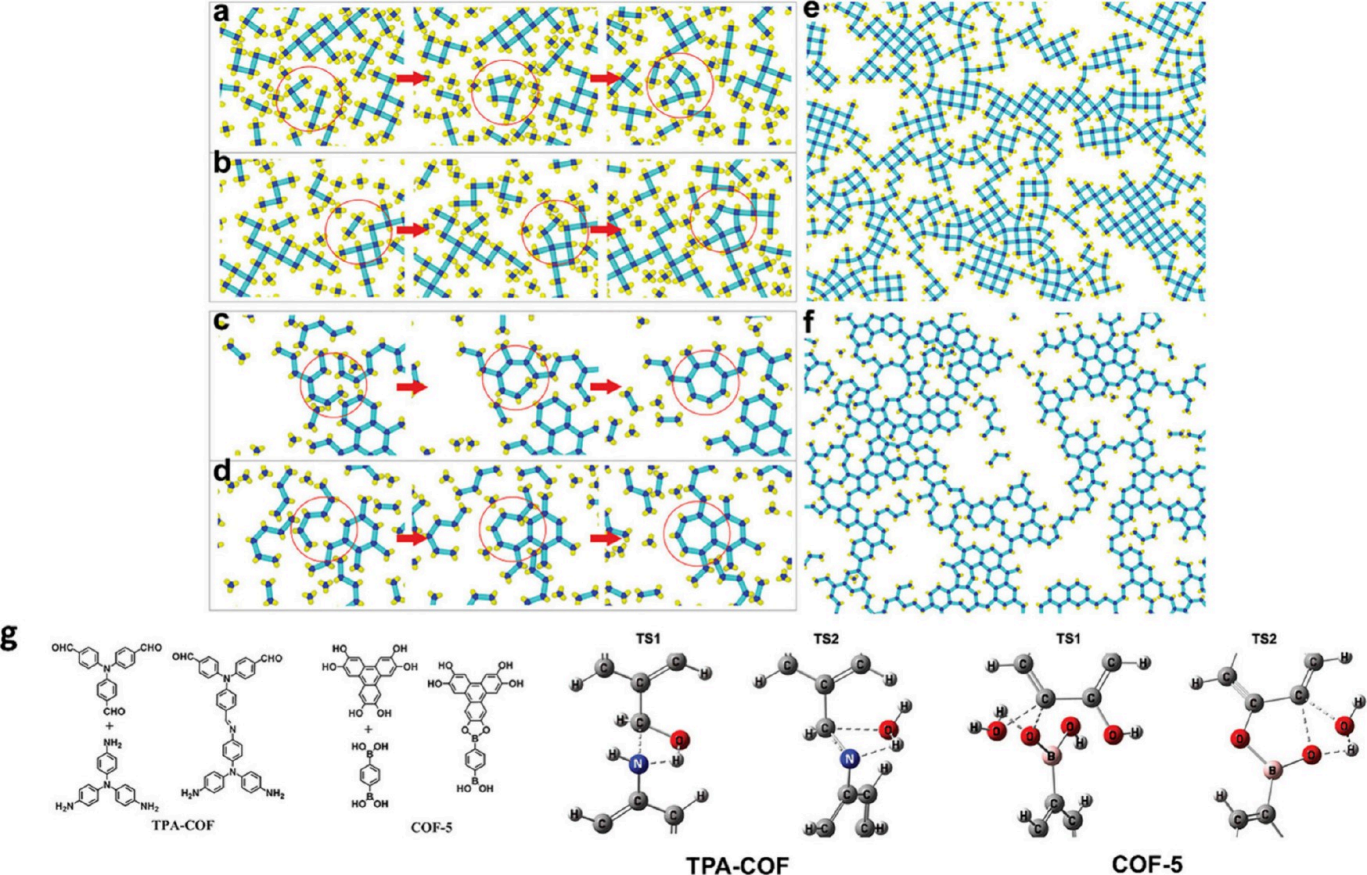
Models of topological defect generation processes for (a and b) [C_4_ + C_4_] with two types of pentagons, and (c and d) [C_3_ + C_3_] with heptagons. The structures formed are (e) [C_4_ + C_4_] and (f) [C_3_ + C_3_]. Schematic synthesis of TPA-COF and COF-5 through the condensation reaction and their possible transition states (g). Reproduced with permission from ref. [^63^]. Copyright 2020 Royal Society of Chemistry.

They also exhibited the importance of building block design in the fabrication of COFs, in which the monomer, dimer, and trimer units form more defects because of their linear growth and hexamers have a tendency to directional uniform growth in polymerization process.^89^

Li et al.^48^ studied computationally the nature of potential defects and their impact on the mechanical properties in borate and imine 2D COFs from classical force fields and MD simulations. Consequently, they simulated standard polymers, and they increasingly created location or line defects across the COF monolayers. The structural defects created in the formation of COF-5 were simulated that the borate COF creation under typical experimental conditions occurred not *via* nucleation but far from equilibrium over spinodal decomposition, causing to a massive defects, which are difficult to correct and form high crystalline COF.^143^ In addition, structural defects in COF-5, as vacancies and grain boundaries, were investigated by MD simulation, leading to considerably larger twists and deformations.^144^ In another work, different stacking arrangements and their effects on the structural and electronic properties in 2D COFs were investigated. Accordingly, the DFT approach showed that the eclipsed stacking (slipped AA-stacking) configurations as the optimal energy because of the stronger interlayer π–π interactions (Figure 19).^39^ However, the statically calculated PXRD patterns showed differences in comparison with experimental results. Instead, MD simulation is in good agreement with experimental results. This work presents the need for accurate modeling of the stacking arrangement in 2D COFs for regulating the COF properties.

**Figure 19:**
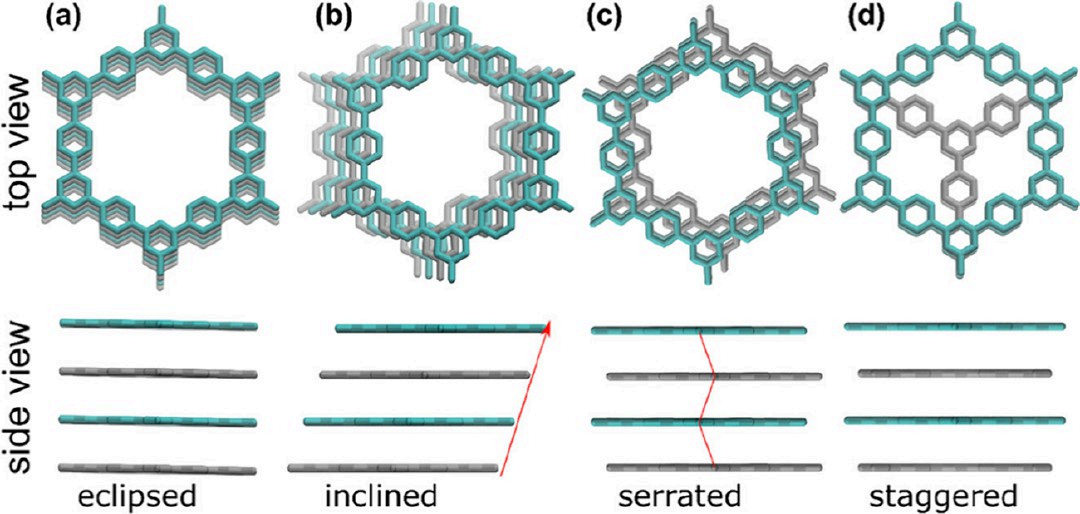
Diverse kinds of stacking in 2D COFs: (a) eclipsed AA-stacking, (b) inclined AA-stacking, (c) serrated AA-stacking, and (d) staggered AB-stacking. Reproduced with permission from ref. 39. Copyright 2022 American Chemical Society.

Although research has focused on the study of COFs mechanisms, the study is delimited to boronate ester- and imine-linked 2D COFs.^145–148^ Therefore, it is highly needed to explore the mechanisms of the other COFs.^149^

## 3 Conclusion

From the previous sections, it becomes clear that there is still not a perfect technique to provide complete information about the density of defective sites in COFs. Some techniques like XRD or gas adsorption give very valid information on structural defects causing loss of crystallinity or variations of surface area and porosity. Some other techniques provide complementary information on the defects consisting of missing monomers and the presence of dangling reactive functional groups. From the above discussions, all the techniques have limitations, and each could be more appropriate in certain cases. Most recently, advanced STEM can visualize defects in COF samples at quasi atomic resolution. However, even this powerful technique has several drawbacks derived from the need for very advanced equipment not available for routine studies and also from the fact that quantification and adequate evaluation of defects require a detailed statistical analysis of different parts of the sample. In other cases, such as acid–base titration using probe molecules, the very valuable quantitative information is limited to examples of COFs having acid or basic sites, and therefore, it lacks universal applicability.

Considering the increasing importance of engineering defects in COFs as a tool to improve performance and increase efficiency, it can be anticipated that defect characterization will gain significant importance in the field of COFs for most applications. This review provides a general overview of how to characterize and quantify these defects.

As the field of COF defect engineering continues to evolve, several key areas for future research and development emerge:

1. Advanced *in situ* characterization: Developing techniques for real-time monitoring of defect formation during COF synthesis. This could involve the use of synchrotron-based X-ray techniques or environmental TEM to observe the growth and defect formation processes in real-time.
2. Defect-property relationships: Gaining further insights on how specific defects influence COF properties and performance in various applications. This includes investigating the impact of defects on mechanical properties, chemical stability, and functional performance in areas such as catalysis, gas storage, sensing, etc.
3. Multiscale modeling: Developing comprehensive computational models that can predict defect formation, distribution, and their impact on COF properties across different length scales, from atomic to macroscopic.
4. In operando studies: Developing techniques to determine defect performance and evolution under realistic operating conditions for various applications, such as during catalysis or gas adsorption/desorption cycles.

Addressing these challenges will require interdisciplinary collaboration and the development of new analytical tools tailored specifically to COF materials. This review is aimed at fostering advancement in the field of COF defect engineering and harnessing these imperfections for enhanced material performance across a wide range of applications.
